# Supplementary material for: Evaluation of Specific Cellular and Humoral Immune Response to Toxoplasma gondii in Patients with Autoimmune Rheumatic Diseases Immunomodulated Due to the Use of TNF Blockers
Source: Biomedicines. 2023 Mar 17;11(3):930. doi: 10.3390/biomedicines11030930 (PMC10046324; doi:10.3390/biomedicines11030930)
Supplement: Supplementary file 1 [file biomedicines-11-00930-s001.zip › biomedicines-1986551-supplementary.pdf]

Supplementary material

Table S1. Description of study groups and control group.

**General features N (%)**			
	**GC (n = 13)**	**DS (n = 14)**	**IB (n = 16)**
**Gender**			
Female	9 (69.2)	12 (85.7)	11 (68.8)
Male	4 (30.8)	2 (14.3)	5 (31.2)
**Age**			
17 – 30	4 (30.8)	0 (0)	1 (6,2)
31 – 45	7 (53.8)	2 (14.3)	5 (31.3)
46 – 60	2 (15.4)	12 (85.7)	10 (62.5)
**Doença autoimune**			
Rheumatoid arthritis	-----	11 (78.6)	7 (43.8)
Rheumatoid arthritis + JIA	-----	1 (7.1)	0 (0)
Rheumatoid arthritis + Crohn's disease	-----	0 (0)	1 (6.2)
Juvenile Idiopathic Arthritis (JIA)	-----	0 (0)	1 (6.2)
Psoriatic arthritis + Psoriasis	-----	1 (7.1)	3 (18.8)
Ankylosing spondylitis	-----	0 (0)	4 (25)
Rheumatoid arthritis + SLE? Or Sjogren (DH)	-----	1 (7.1)	0 (0)

SLE = Systemic Lupus Erythematosus; DH = Diagnostic hypothesis.

Table S2. Description of drugs in groups IB and DS.

	**Time of use**						
**Biological immunomodulators (n = 16)**	**Up to 3 months**	**4 to 6 months**	**7 to 12 months**	**13 to 24 months**	**25 to 60 months**	**61 to 120 months**	**More than 120 months**
Adalimumabe				2			1
Adalimumabe, Leflunomide e PDN					1		
Certolizumabe					1		
Certolizumabe e PDN		1					
Certolizumabe, HQN e PDN					1		
Certolizumbe, MTX, Mesalazine e Arpadol					1		
Etanercepte					2	1	
Etanercepte e MTX						1	
Golimumabe e MTX					1		
Infliximabe e MTX				1		1	
Infliximabe e PDN					1		
**Synthetic drugs* (n = 14)**							
Corticosteroids (Dexamethasone a ou Prednisone)		2					
MTX				1	2		
MTX e Hydroxychloroquine					1		
MTX, Hydroxychloroquine e Prednisone		1			4		
MTX e Leflunomide						1	
MTX e Prednisone					2		

* PDN = prednisone; HQN = hydroxychloroquine; MTX = methotrexate.

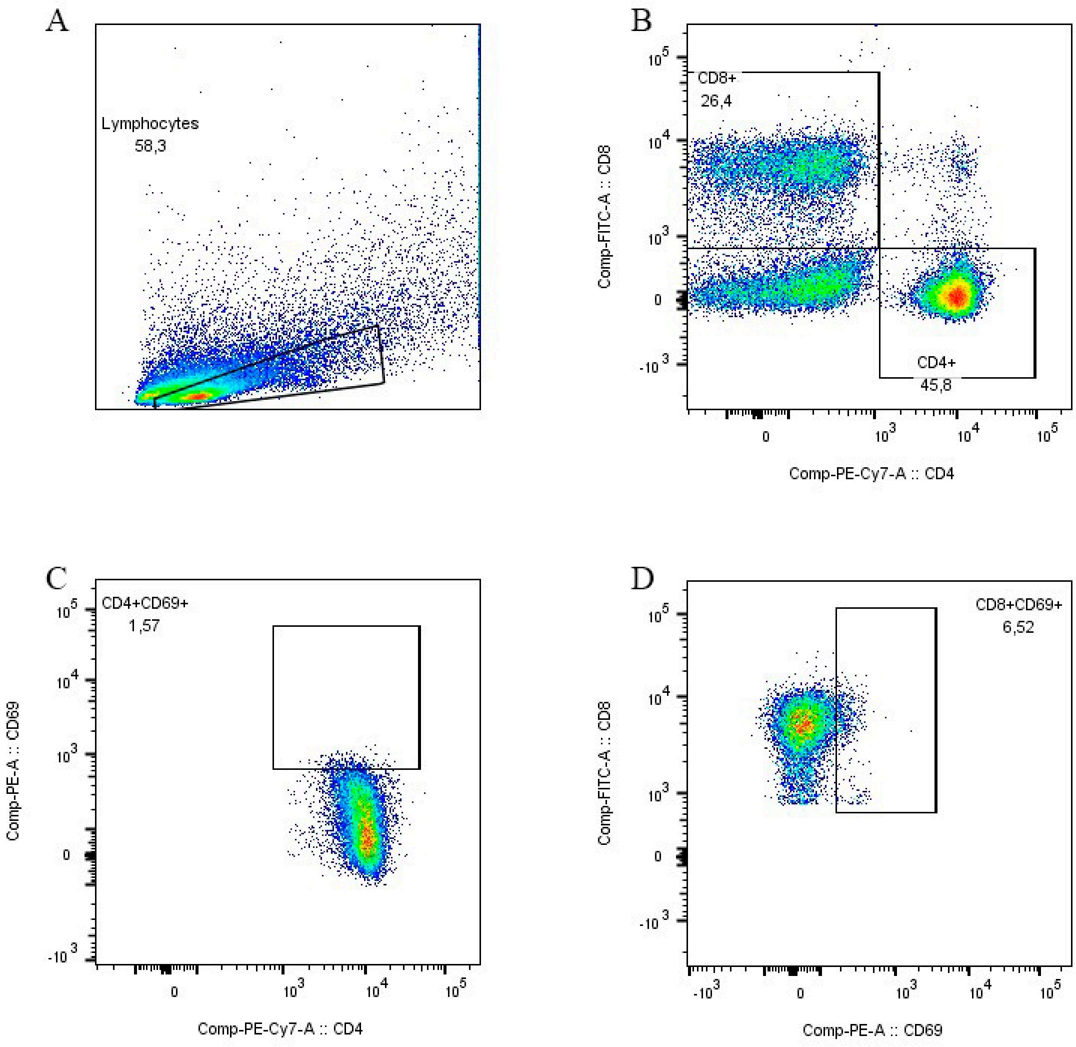

Figure S1 – Demonstration of the selection of gates for analysis of the acquisition of CD4+ and CD8+ T lymphocytes activated early, evaluated by the expression of cell surface receptor CD69. (A) Selection of total lymphocyte population, (B) selection of CD4+ and CD8+ T cell populations, among total lymphocyte populations, (C) selection of CD4+ cells labeled with CD69+, among total CD4+ cells, and (D) selection of CD8+ cells labeled with CD69+ from among total CD8+ cells. The numbers presented within the graphs demonstrate the percentage of cells present in each described selection.

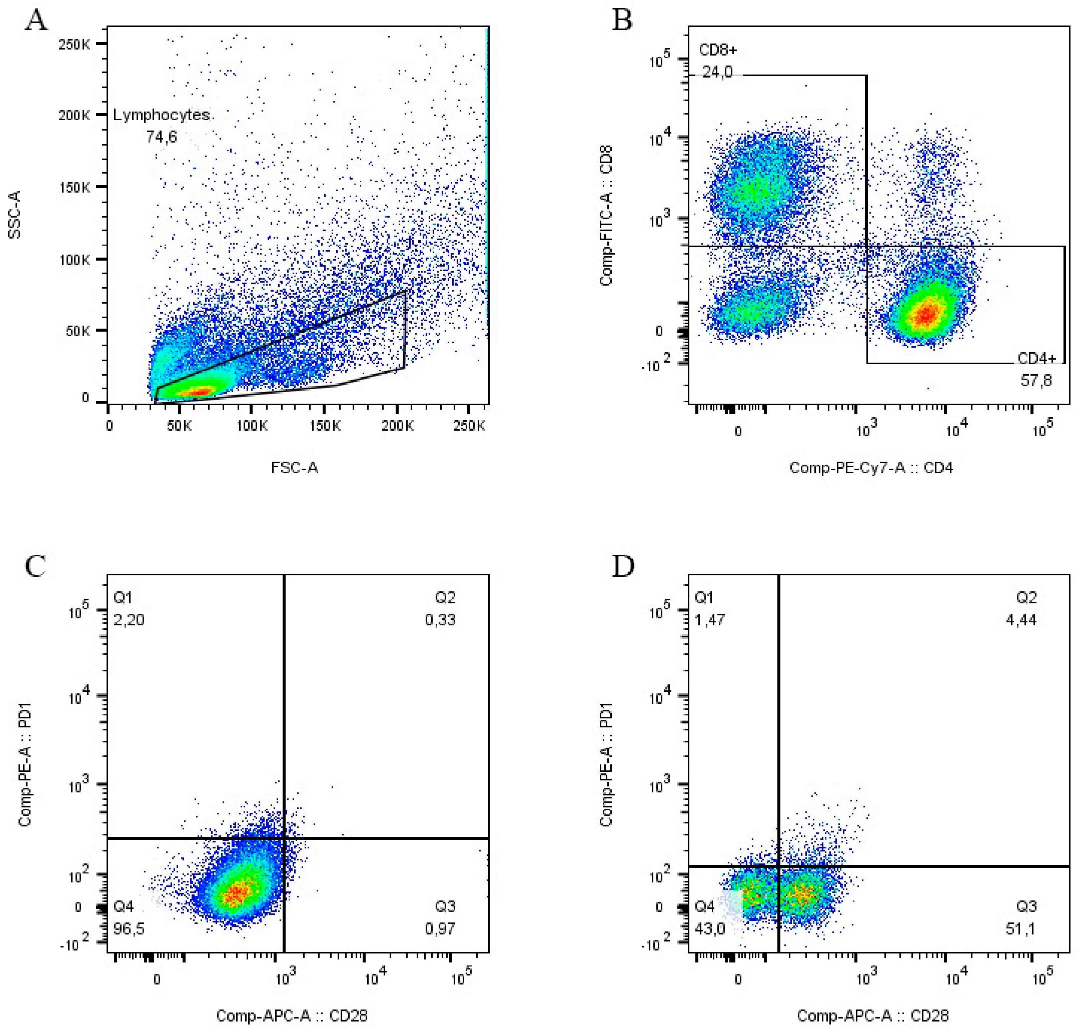

Figure S2 – Demonstration of the selection of gates for analysis of the acquisition of CD4+ and CD8+ T lymphocytes with activation and/or exhaustion, evaluated by the expression of cell surface receptors CD28 and PD-1. (A) Selection of the population of total lymphocytes, (B) selection of populations of CD4+ and CD8+ T cells, among the populations of total lymphocytes, (C) selection of CD4+ cells labeled with PD-1 (Q1 + Q2), CD28+ (Q2 + Q3) and double-labeled with PD-1 and CD28+ (Q2) and (D) selection of CD8+ cells labeled with PD-1 (Q1 + Q2), CD28+ (Q2 + Q3) and double-labeled with PD-1 and CD28+ (Q2). The numbers presented within the graphs demonstrate the percentage of cells present in each described selection.

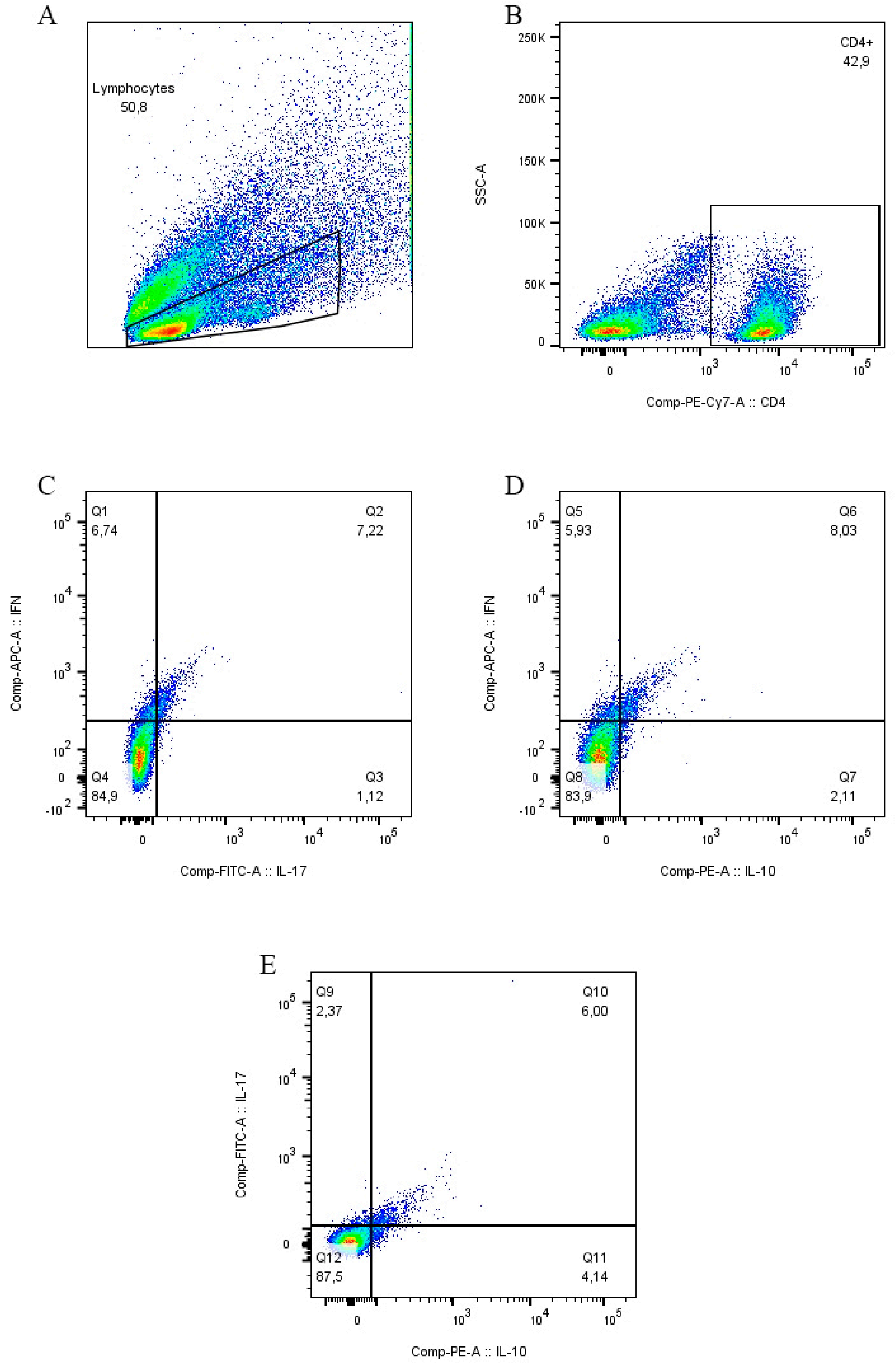

Figure S3 – Demonstration of the selection of gates for analysis of the acquisition of CD4+ T lymphocyte profiles, evaluated by the intracellular expression of the cytokines IFN-γ, IL-17 and IL-10. (A) Selection of the population of total lymphocytes, (B) selection of populations of CD4+ T cells, among the populations of total lymphocytes, (C) selection of CD4+ cells expressing IFN-γ (Q1 + Q2), IL-17 (Q2 + Q3) and double-labeled with IFN-γ and IL-17 (Q2), (D) selection of CD4+ cells expressing IFN-γ (Q5 + Q6), IL-10 (Q6 + Q7) and double-labeled with IFN-γ and IL-10 (Q6) and (E) selection of CD4+ cells expressing IL-17 (Q9 + Q10), IL-10 (Q10 + Q11) and double-labeled with IL-17 and IL-10 (Q10). The numbers presented within the graphs demonstrate the percentage of cells present in each described selection.

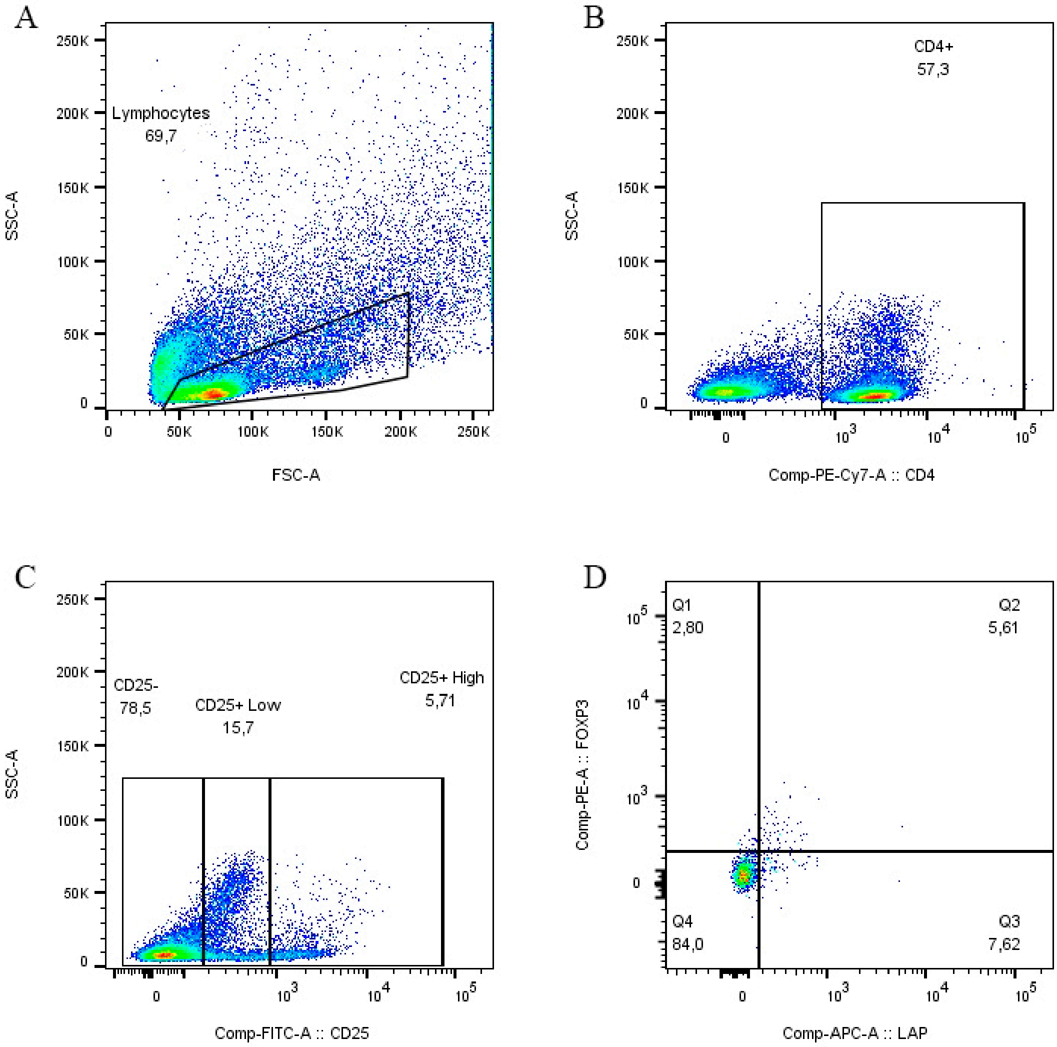

Figure S4 – Demonstration of the selection of gates for analysis of the profile acquisition of regulatory CD4+ T lymphocytes, evaluated by the expression of the alpha chain of the IL-2 receptor (CD25), LAP and the transcription factor Foxp3. (A) Selection of the total lymphocyte population, (B) selection of CD4+ T cell populations, among the total lymphocyte populations, (C) selection of CD25-, CD25Low and CD25High cells, among the CD4+ cell populations and (D) selection of CD4+CD25High cells, expressing FOXP3 (Q1 + Q2), LAP (Q2 + Q3) and doubly labeled with FOXP3 and LAP (Q2). The numbers presented within the graphs demonstrate the percentage of cells present in each described selection.
